# Selecting the best stable isotope mixing model to estimate grizzly bear diets in the Greater Yellowstone Ecosystem

**DOI:** 10.1371/journal.pone.0174903

**Published:** 2017-05-11

**Authors:** John B. Hopkins, Jake M. Ferguson, Daniel B. Tyers, Carolyn M. Kurle

**Affiliations:** 1 School of Biodiversity Conservation, Unity College, Unity, Maine, United States of America; 2 Division of Biological Sciences, Ecology, Behavior, and Evolution Section, University of California San Diego, La Jolla, California, United States of America; 3 Center for Modeling Complex Interactions, University of Idaho, Moscow, Idaho, United States of America; 4 United States Forest Service, Northern Rocky Mountain Science Center, Bozeman, Montana, United States of America; Estacion Experimental de Zonas Áridas (CSIC), SPAIN

## Abstract

Past research indicates that whitebark pine seeds are a critical food source for Threatened grizzly bears (*Ursus arctos*) in the Greater Yellowstone Ecosystem (GYE). In recent decades, whitebark pine forests have declined markedly due to pine beetle infestation, invasive blister rust, and landscape-level fires. To date, no study has reliably estimated the contribution of whitebark pine seeds to the diets of grizzlies through time. We used stable isotope ratios (expressed as *δ*^13^C, *δ*^15^N, and *δ*^34^S values) measured in grizzly bear hair and their major food sources to estimate the diets of grizzlies sampled in Cooke City Basin, Montana. We found that stable isotope mixing models that included different combinations of stable isotope values for bears and their foods generated similar proportional dietary contributions. Estimates generated by our top model suggest that whitebark pine seeds (35±10%) and other plant foods (56±10%) were more important than meat (9±8%) to grizzly bears sampled in the study area. Stable isotope values measured in bear hair collected elsewhere in the GYE and North America support our conclusions about plant-based foraging. We recommend that researchers consider model selection when estimating the diets of animals using stable isotope mixing models. We also urge researchers to use the new statistical framework described here to estimate the dietary responses of grizzlies to declines in whitebark pine seeds and other important food sources through time in the GYE (e.g., cutthroat trout), as such information could be useful in predicting how the population will adapt to future environmental change.

## Introduction

In the Greater Yellowstone Ecosystem (GYE), Threatened grizzly bears (*Ursus arctos*) forage for a wide variety of foods [1] and their individual diets vary depending on their age class, sex, year, and reproductive status [2,3]. It is well understood that many grizzlies forage for plants, ungulates, and whitebark pine (*Pinus albicaulis*) seeds in the GYE [1,4]. It is also recognized that some bears foraged for cutthroat trout (*Oncorhynchus clarki*) when they were abundant in the Yellowstone Lake watershed [5] and that some bears currently specialize on army cutworm moths (*Euxoa auxiliaris*) in the eastern region of the GYE [6]. Unlike other major food sources, past research suggests that grizzlies are better off when whitebark pine crops boom in the GYE. For instance, grizzly bear mortality increases during years of low whitebark pine productivity [7], whereas survival [8] and reproduction increases for independent bears following good mast years [2,3].

Currently, whitebark pine trees are listed as Endangered by the IUCN and as a high priority Candidate Species under the U.S. Endangered Species Act. These designations resulted from massive die-offs in western North America caused by severe infestations of mountain pine beetles (*Dendroctonus ponderosae*), which were exacerbated partly due to climate warming [9], and from infection caused by invasive white pine blister rust (*Cronartium ribicola*) [10]. Understanding how bears have responded to the losses of whitebark pine seeds (and other major food sources such as cutthroat trout) has been a conservation priority since the delisting of the Yellowstone grizzly population was vacated in September 2009 [11]. The main reason the Threatened status of the grizzly was reinstated in March 2010 was because it was unclear how declining whitebark pine will impact long-term population trends [12].

Felicetti et al. [13] and Schwartz et al. [14] used stable isotopes to investigate the consumption of nutrient-rich whitebark pine seeds by Yellowstone grizzlies. Felicetti et al. [13] had two main objectives: i) to model the relationship between *δ*^34^S values (believed to be an indicator of whitebark seed consumption) derived from grizzly tissues (hair and blood) and cone production (mean number of cones per tree); and ii) to estimate the contribution of whitebark pine seeds to the diets of grizzly bears using *δ*^34^S and *δ*^15^N values (the latter being a relative measure of protein consumption; [15]). For objective 1, they found that the mean annual *δ*^34^S values for grizzlies increased with cone production (suggesting higher resource use), except for one year (2000) of seven in which *δ*^34^S values were relatively high for bears and cone production was low. They explained that high cone production in 1999 likely caused bears to forage heavily on overwintered masts in the spring and summer of 2000, increasing *δ*^34^S values for bears that year. For objective 2, they estimated that 11–100% of bears’ assimilated diets were composed of whitebark pine seeds, depending on year [13]. They stated that unique solutions for dietary proportions were impossible given their 2-isotope, 5-source model, acknowledging that the sources in their isotopic mixing space (the area or volume contained in the space formed by lines connecting the sources in a multivariate plot of isotope values) were too numerous (a system with more than *n+1* sources, where *n* is the number of isotope tracers, does not have a unique, deterministic solution) to obtain unique solutions. They also showed that some isotope values for these sources were collinear (lying along the same straight line) with those for bears, leading to non-identifiable solutions. Recently, Schwartz et al. [14] conducted a partial reassessment of objective 1 from Felicetti et al. [13] and found similar results for the relationship between the *δ*^34^S values for grizzly hair and cone production, but reported that other bear foods may have similar *δ*^34^S signatures as whitebark pine seeds, confounding any results generated by stable isotope mixing models (hereafter, SIMMs).

Even though there have been considerable advancements in modeling the diets of omnivores using SIMMs over the past decade [16], no study since Felicetti et al. [13] has attempted to quantify the contribution of whitebark pine seeds (which if conducted correctly would account for any overlap in stable isotope values of food sources) to the diets of grizzlies in the GYE using SIMMs. In this study, we revisited objective 2 in Felicetti et al. [13] using modern SIMM analysis and a novel model selection framework (Box 1).

**Box 1. Estimating the diets of grizzly bears in Cooke City using a 4-step approach for modeling the diets of animals using stable isotope analysis.**

(1) ***Plotting stable isotope data corrected for isotopic discrimination.*** It is important to use accurate isotopic discrimination factors when estimating the assimilated diets of free-ranging animals using stable isotope analysis (hereafter, SIA) [28]. We added isotopic discrimination factors (small offsets of stable isotope values between dietary sources and animal tissues due to metabolic and digestive processes; expressed as *∆*^13^C, *∆*^15^N, *∆*^34^S) for omnivorous rats [15,29] (laboratory rats, *Rattus norvegicus*) to the isotope values of each major food source (Fig 2; S2 Table). Correcting the stable isotope values for each sampled food item in such a manner allowed us to relate the isotopes in food items to those measured in bear hair. We used discrimination factors for hair derived from laboratory rats because no study has accurately estimated the discrimination factors for bear hair. Because grizzly bears and rats are monogastric mammalian omnivores, we assumed grizzlies discriminate against ^12^C, ^14^N, and ^32^S by a similar magnitude as rats [30]. We added discrimination factors for laboratory rats held on known diets composed of either plants (wheat: *∆*^13^C = 3.4±0.5 ‰; *∆*^15^N = 2.4±0.2 ‰) or animals (fish: *∆*^13^C = 2.1±0.1 ‰; *∆*^15^N = 3.9±0.3 ‰) [29] to the *δ*^13^C and *δ*^15^N values for plants and animals used in this study (Fig 2; S2 Table). We note that *∆*^13^C for rat hair ($\bar{x}$ = 2.9±0.9 ‰; [29]) was similar to other mammals ($\bar{x}$ = 2.5±0.9 ‰; [31]). We also added sulphur discrimination factors (*∆*^34^S) for laboratory rats calculated from a regression equation in Florin et al. [15] to *δ*^34^S values for all sampled foods (Fig 2; S2 Table).

We plotted isotope values for bear hair and discrimination-corrected isotope values for the three main bear foods in Cooke City using 4 multivariate plots (Fig 2). Hereafter these combinations of stable isotope values are denoted as *δ*^13^C/*δ*^15^N, *δ*^34^S/*δ*^15^N, *δ*^13^C/*δ*^34^S, and *δ*^13^C/*δ*^15^N/*δ*^34^S.

(2) ***Correcting the mixing space for digestible elemental concentrations and assessing collinearity between mixtures and sources***. Not accounting for differences in digestible elemental concentrations among food sources can bias dietary estimates calculated by SIMMs [32]. We calculated the relative differences in stoichiometry and differential digestibility of C and N (S2 Table). We calculated digestible [C] and [N] using data from past studies [33] or comparable food items from the USDA Nutrient Database (www.nal.usda.gov/fnic/foodcomp/search; S2 Table). We held digestible S fixed at 1 for all food sources in our analysis because it is currently unclear how bears or other mammals digest S. We assumed that if estimated dietary contributions for the population were similar between models with and without S, our fixed value for digestible [S] was reasonable to use in our study.

Collinearity among sources and mixtures in an isotopic mixing space (corrected for isotopic discrimination and digestible elemental concentrations) results in multiple solutions for dietary contributions that are statistically equivalent. We assessed the geometry of each mixing space and assumed that those showing signs of collinearity would not converge or yield estimates that were consistent with those generated by other combinations of stable isotope measurements.

(3) ***Fitting SIMMs to stable isotope data for consumers*.** We initially ran four models in each candidate set that estimated the diets of bears at the individual-level (as a random effect), as we assumed diet heterogeneity exists in the population. Each candidate set was designated as such based on a different 2- or 3-isotope combination of *δ*^13^C, *δ*^15^N, and *δ*^34^S values derived from bear hair (the responses). Each candidate set contained a null model, which included stable isotope values but did not include digestible elemental concentrations ([C], [N], [S]) for each food source (concentration dependence), and a series of models that included stable isotope values and incorporated concentration dependence. We developed a notation for SIMMs in order to facilitate the comparison of the wide array of models tested in this work. Our notation utilizes a similar structure to that used in generalized linear models in R [34], which are familiar to many ecologists. Models are of the form Response(.)~Covariate. Response corresponds to the combination of isotope tracers used (e.g., CNS for a model that uses *δ*^13^C, *δ*^15^N, and *δ*^34^S values), and its argument can be used to note any modifications to the mixing model such as the inclusion of concentration dependence (CD). The right-hand side of the model formula is used to denote the covariates used to model diet proportions. For example, CN ~ (1|BearID) is a null model that uses *δ*^13^C and *δ*^15^N values to estimate dietary proportions for each individual bear (as a random factor).

In addition to the null model, each candidate set was composed of a set of concentration dependence models with and without group structure (defined as a set of observations that share a common property and estimated in hierarchical models). We considered sex and the year that bears were sampled as two different groups. In addition to bear ID, we treated these groups as random effects in our models. Hereafter, we refer to models that include concentration dependence with group structure (sex or year) and those without these random effects as our three “main” models. We did not include an interaction between sex and years because all male bears were sampled in one year (2009) and did not include additional levels of hierarchy (sex and years as random effects in the same model) because our sample was small. We expected that the null model would fit the data poorly compared to other models in each candidate set because this model did not take into account concentration dependence [32]. After running our initial set of four models, we included cutthroat trout in a 4-source model (plants, ungulates, whitebark, cutthroat trout), using the same model structure as the top model (described below in step 4) in each candidate set. We included these additional models in our study to demonstrate an approach for comparing models that account for different numbers of sources. Such analyses could be useful to ecologists for identifying a “potential” food source that is important (e.g., trout) to animals. We also included trout to confirm they were not contributing to bear diets in 2007–2009, to validate the accuracy of our models (i.e., model prediction of trout contribution to diet = 0%), and to provide a model to estimate the diets of bears through time in the Yellowstone Lake watershed.

We used IsotopeR [35] to estimate the mean proportional dietary contributions for bears at the population-, group- (sex or years when applicable), and individual-level. We applied measurement error to each sample based on results from the Colorado Plateau lab (*δ*^13^C±0.2 ‰, *δ*^15^N±0.3 ‰, and *δ*^34^S±0.4 ‰), as these estimates of error were higher, and therefore more conservative, than those from the Washington State lab. For all models, we applied discrimination error (SD associated with discrimination factors [15,29]); used uninformative priors; and ran three MCMC chains, a burn-in of 10^3^ draws, followed by 10^4^ draws from the posterior. We reported the mean, 1 SD, median, and 95% credible interval (CI) for each mean marginal posterior density distribution (i.e., proportional dietary contribution) for each major food source. We combined chains by concatenation to calculate summary statistics. We calculated rhat (not reported) and DIC values following the calculations in R’s CODA package [36] and in the sampling software, JAGS (described in [37]).

(4) ***Assessing goodness of fit and relative support for competing models*.** We calculated the goodness of fit of each stable isotope tracer in each SIMM model and conducted model selection within and between candidate sets. We used IsotopeR to estimate proportional dietary contributions for bears using each model. We then compared the goodness of fit of each isotope tracer in each model by calculating the normalized root-mean-squared error (NRMSE),

$${NRMSE}_{s}=\frac{{RMSE}_{s}}{Max(y_{s})-Min(y_{s})}=\frac{\left(\sqrt{\frac{\sum_{i=1}^{n_{s}}{\left({\hat{y}}_{si}-y_{si}\right)}^{2}}{n_{s}}}\right)}{Max(y_{s})-Min(y_{s})}$$ (1)

where there are *n*_*s*_ values of the estimated (${\hat{y}}_{si}$) and observed (*y*_*si*_) stable isotope ratios for tracer *s* and consumer *i*. Each NRMSE_s_ (Eq 1) is a standardized sample standard error. A relatively low NRMSE suggests relatively less residual variance for *s* and thus a better fit than other stable isotope tracers. This statistic allowed us to determine if certain stable isotope tracers had potentially more predictive power than others despite their different scales.

We used the deviance information criterion (DIC) to select the best model in each candidate set because it is widely used for Bayesian model selection. The DIC is composed of a goodness of fit term (the expected deviance: $\bar{D}$) and a complexity term (the effective number of parameters: *p*_*D*_). The latter term penalizes the deviance as the number of parameters increase. We also reported the penalized expected deviance (PED) for each model because this information criterion might be better suited than DIC for evaluating hierarchical mixture models [38]. We assumed model selection within a candidate set will have the most applicability to other diet studies because it is common for ecologists to measure two isotope ratios in consumer and prey tissues.

We also rescaled the deviance and recalculated DIC (Eq 2) to compare models with the same model structure (hereafter, cohort models) between candidate sets with different sample sizes. We recalculated DIC (*DIC*_*rescaled*_) for each model by rescaling the expected deviance ($\bar{D}$) estimated under the sample size (*n*_*sample*_) to a new sample size (*n*_*rescale*_):

$${DIC}_{rescaled}=\frac{n_{rescaled}}{n_{sample}}\bar{D}+p_{D}$$

We used the DIC_rescaled_ metric to compare 2- (*n*_*sample*_ = 34) and 3-isotope (*n*_*sample*_ = 51) models under the hypothetical scenario that they have the same amount of data (i.e., nrescale = 34). We used this calculation to determine whether future work will benefit from collecting additional stable isotope data (3- vs. 2-isotope models).

We reported the proportional dietary contributions for top models in each candidate set (lowest DIC), which includes the top model overall (lowest DIC_rescaled_). We also estimated the probability of similarity (defined as the probability that two proportional dietary contributions are the same; i.e., the lower the probability of similarity, the greater the difference between diet contributions; [39]) for both males and females and between years for the highest ranking sex and year models, respectively, and used this method to confirm that models within the same cohort generated similar proportional dietary contributions.

Surprisingly, no effort has been dedicated to developing an approach for model selection to compare alternative SIMMs. In particular, no studies to our knowledge have tested competing models that use different isotopic mixing spaces (the area or volume contained in the space formed by lines connecting the sources in a multivariate plot of isotope values) to estimate the dietary contributions of consumers. Such analyses are particularly important for modeling the diets of omnivores, as subtle changes to an isotopic mixing space due to the inclusion or exclusion of covariates (i.e., different hypotheses that explain assimilated diets) can have an effect on the proportional dietary contributions estimated by SIMMs.

The main purpose of our study was to demonstrate the utility of model selection for SIMM analysis by estimating the diets of grizzly bears in Cooke City Basin, Montana (Box 1). We first sampled grizzlies using hair-snares and identified each individual using microsatellite genotyping. We then used stable isotope ratios (expressed as *δ*^13^C, *δ*^15^N, and *δ*^34^S values) derived from the hair of each individual bear and their foods to estimate the proportional contributions of major food sources to the diets of grizzlies sampled in Cooke City Basin in 2007–2009. We conducted our analyses using 4 sets of SIMM models. Each candidate set was defined by a different combination of stable isotope values from grizzly bear hair and their foods. We compared *δ*^13^C, *δ*^15^N, and *δ*^34^S values from bear tissues through time and tested the explanatory power of each stable isotope tracer in each model using a goodness of fit measure. We then conducted model selection within and among candidate sets to determine the best models for estimating the diets of grizzly bears. In the end, we provide a modeling strategy to estimate the grizzly bear diets through time in the GYE using stable isotopes.

## Materials and methods

### Study area

**Fig 1:**
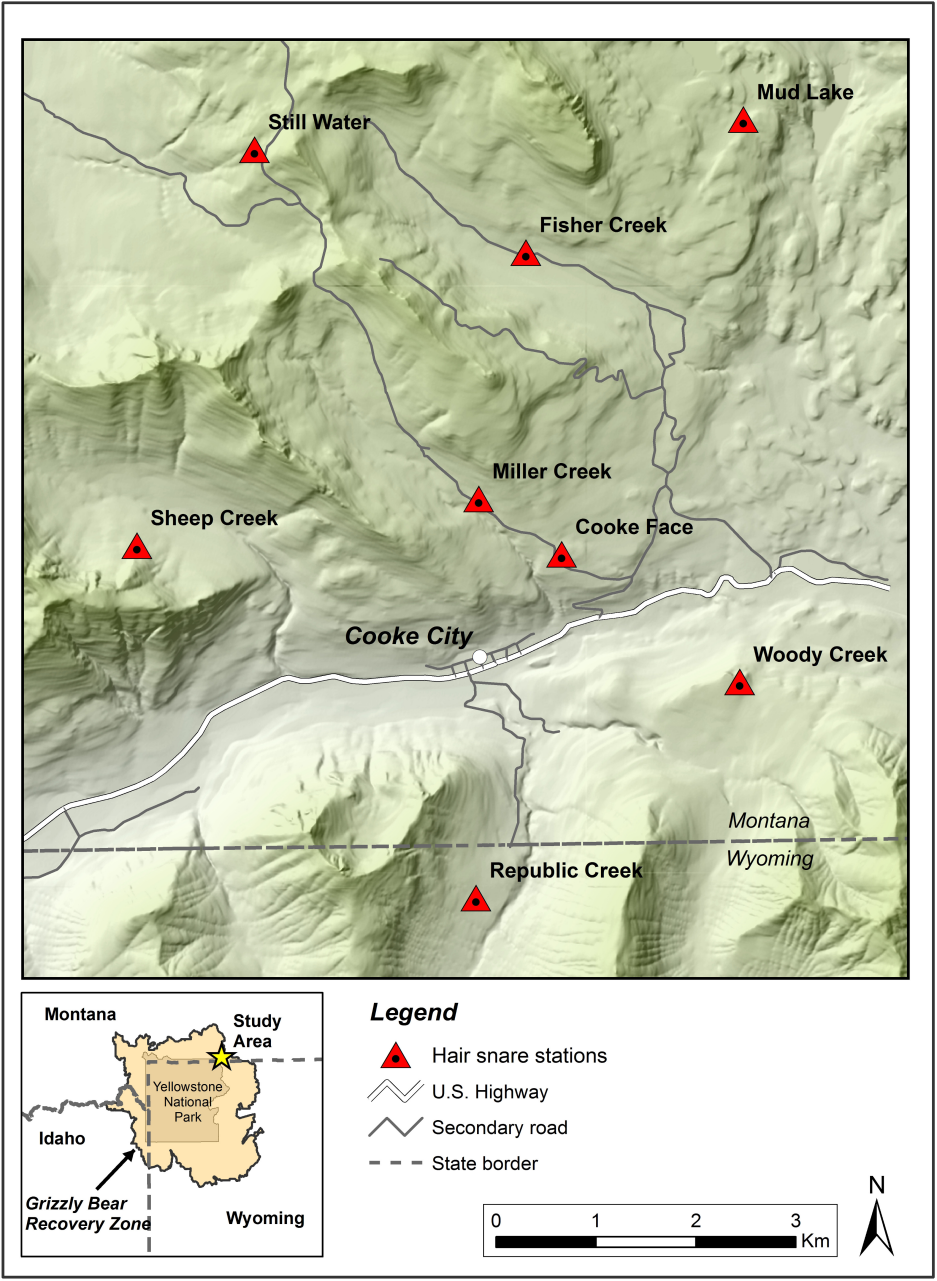
Locations of hair-snares in Cooke City Basin, Montana, 2007–2009.

Cooke City Basin is located northeast of Yellowstone National Park within the Grizzly Bear Recovery Zone (U.S. Fish and Wildlife Service 1993) (Fig 1). Our study area was an ideal location for this investigation because Cook City Basin is central to the largest expanse of whitebark pine forests comparatively unaffected by mountain pine beetle in the GYE. The study area was divided into eight subunits, as determined by mountain configuration and drainages (Fig 1).

The vegetation in the study area is a mosaic of forested community types fragmented by talus, avalanche chutes, non-forested openings, and alpine meadows. Forested areas in Cooke City Basin may contain Engelmann spruce (*Picea engelmannii*), whitebark pine (*Pinus albicaulis*), lodgepole pine (*Pinus contorta*), and/or subalpine fir (*Abies lasiocarpa*). Like much of the GYE, the Cooke City Basin supports a diversity of large ungulates, including elk (*Cervus elaphus*), bison (*Bison bison*), mule deer (*Odocoileus hemionus*), white-tailed deer (*O*. *virginianus*), and moose (*Alces alces*).

### Sampling

#### Grizzly bears

The following monitoring protocol was part of a much larger effort designed to assess the relationship between grizzly bear habitat use in the Cooke City Basin and human activities. The protocol was developed through the formal consultation process of the U.S. Fish and Wildlife Service and U.S. Forest Service, as prescribed under Section 7 of the Endangered Species Act. We sampled grizzlies in each major drainage in Cooke City Basin, Montana (Fig 1 and S1 Table). We positioned eight hair-snares along game trails and other travel corridors to maximize capture of grizzly hair (S1 Table). In 2007 and 2008, we collected bear hair from creosote-impregnated planks that were nailed to trees. In 2009, we used a modified method to collect bear hair [17]. We strung 30 m of 4-prong, 2-strand barbed wire around ≥3 trees. To reduce the probability of capturing young bears, we strung barbed wire ~50 cm from the ground. We targeted independent bears in 2009 because the assimilation of stable isotopes from milk can increase the *δ*^15^N values measured in the tissues of nursing grizzly bears [18]. We baited stations in 2009 with anise oil by pouring the liquid over each plank centered in the trap. We visited hair-snares every two weeks in July–October to collect hair samples and add new lure. At inspection, each barb with hair was considered a separate sample. We inserted each sample into a paper envelope and stored all samples in a desiccant chamber.

Hair samples represented the diets of bears in 2007–2009 (Table 1). We assumed that stable isotopes derived from full-length guard hairs collected in September and October represented bear diets during the year they were sampled. We considered stable isotope values for hair from recaptured bears in consecutive years as independent in our analysis.

**Table 1:** Stable isotope values (*δ*^15^N, *δ*^34^S, *δ*^13^C) (‰) for grizzly bears sampled in Cooke City Basin, Montana, 2007–2009.

ID	Sex	Year	*δ*^13^C	*δ*^15^N	*δ*^34^S
071	F	2008	-21.5	5.1	2.8
073	F	2007	-21.5	1.5	5.0
073	F	2008	-22.7	1.5	3.8
073	F	2009	-23.6	1.4	2.3
081	F	2009	-23.1	2.3	4.1
086	F	2008	-22.8	3.8	3.3
089	F	2009	-23.1	2.5	3.5
092	F	2008	-22.3	2.8	4.4
106	F	2007	-22.0	2.4	5.4
108	F	2007	-22.3	4.5	5.1
108	F	2009	-22.5	3.9	3.4
09–151	F	2009	-23.0	3.6	3.2
09–290	F	2009	-23.1	3.8	3.4
Mean—Female			-22.6	3.0	3.8
1 SD			0.6	1.2	0.9
09–164	M	2009	-23.3	2.1	3.4
09–228	M	2009	-23.5	3.4	0.0
09–474	M	2009	-22.7	4.3	2.7
116277	M	2009	-22.5	2.3	2.8
Mean—Male			-23.0	3.0	2.2
1 SD			0.5	1.0	1.5
Mean–All			-22.7	3.1	3.5
1 SD			0.6	1.1	1.3
		2007			
Mean			-21.9	2.8	5.2
1 SD			0.4	1.5	0.2
		2008			
Mean			-22.3	3.3	3.6
1 SD			0.6	1.5	0.7
		2009			
Mean			-23.0	3.0	2.9
1 SD			0.4	1.0	1.1
Between male and females:					
*δ*^13^C: *t* = 1.424, *df* = 6.749, *P* = 0.199					
*δ*^15^N: *t* = -0.028, *df* = 5.815, *P* = 0.978					
*δ*^34^S: *t* = 1.997, *df* = 3.725, *P* = 0.122					
Among years:					
*δ*^13^C: *F(1*,*15)* = 18.54, *df* = 1, *P*<0.005 (ANOVA)					
*δ*^15^N: *F(1*,*15)* = 0.001, *df* = 11, *P* = 0.975 (ANOVA)					
*δ*^34^S: *H* = 7.82, *df* = 2, *P* = 0.02 (Kruskal–Wallis)					

#### Grizzly bear foods

**Fig 2:**
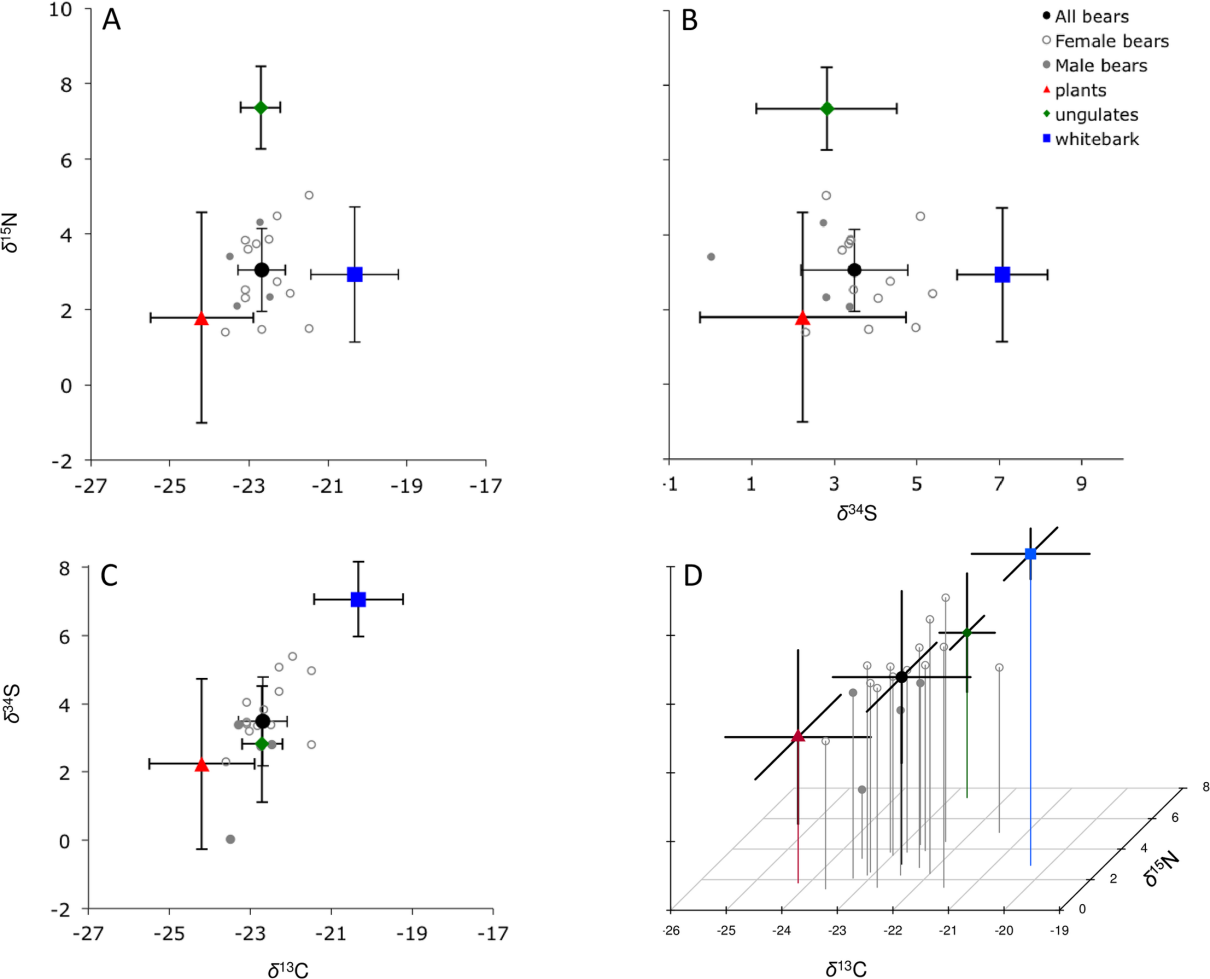
Stable isotope values (*δ*^13^C, *δ*^15^N, *δ*^34^S) (‰) for grizzly bear hair and major bear foods (corrected for isotopic discrimination) in Cooke City Basin, Montana, 2007–2009.

We primarily used stable isotope values for the main food sources (defined as major contributors to assimilated biomass) available to grizzlies in Cooke City: plants, ungulates, and whitebark pine seeds (Fig 2 and S2 Table). We used stable isotope values derived from plant and animal tissues collected from GPS site visits where evidence of feeding by grizzlies was detected, and stable isotope values for whitebark pine seeds collected in National Forests in the Greater Yellowstone-Grand Teton seed zone [19,20]. We did not include army cutworm moths in our analysis because bears do not forage for this food source near our study area [6].

Although spawning cutthroat trout (*Oncorhynchus clarkii*) were historically available to bears as prey in the Yellowstone Lake watershed, introductions of whirling disease (*Myxoblus cerebralis*) and non-native lake trout (*Salvelinus namaycush*) severely reduced their numbers over the past few decades [5]. Like moths, we assumed cutthroat trout were not a major food source for bears in Cooke City Basin during the course of this study [1,19].

### Analytical procedures

#### Genetic analysis

We used microsatellite genotyping to identify individuals, then we sent hair samples to Wildlife Genetics International (WGI; Nelson, British Columbia, Canada) to identify individuals. Before sending the hair, we sub-selected 333 samples to reduce the probability of identifying the same individual multiple times each survey [21]. WGI extracted DNA from the roots of hairs using QIAGEN’s DNeasy Blood and Tissue kits (Qiagen, Mississiauga, Ontario, Canada), following the manufacturer’s instructions. WGI used 10 guard hair roots, when available, to reduce the probability of genotyping errors [22]. For 2007 and 2008 samples, WGI first removed low quality samples using a single-locus prescreen (G10J), then used 9 additional microsatellite markers (G1D, G10B, G10H, G10J, G10L, G10P, MU23, MU51, and MU59) to identify individual grizzly and black bears [23]; they used the full 10-locus system to identify individuals in 2009. Only samples that satisfied a series of strength and appearance criterion at each of the 10 markers were considered to be genotyped successfully. WGI used the amelogenin system to determine the sex of each bear in all years [24]. In the end, WGI identified and corrected genotyping errors by reanalyzing the mismatching markers in pairs of genotypes that were similar enough to raise concerns (i.e., those that mismatch at just 1 or 2 markers) [25]; this protocol has been shown to effectively prevent the recognition of false individuals through genotyping errors [26].

#### Stable isotope analysis

We measured the abundances of stable isotopes in the hair for each bear. Washington State University prepared and analyzed all samples as described in Felicetti et al. [13]. We rinsed bear hair with a 2:1 chloroform-methanol solution to remove surface oils, then air-dried hair for 24 hours. We weighed and sealed all samples (0.9–4.1 mg) into 5x9 mm tin capsules (Costech Analytical Technologies, Inc., Valencia, CA, USA).

Washington State University Stable Isotope Core Laboratory measured the abundances of carbon, nitrogen, and sulfur stable isotopes in bear foods using a ECS 410 (Costech Analytical, Valencie, CA, USA) elemental analyzer interfaced with a Delta Plus XP (Thermo-Finnigan, Breman, Germany) mass spectrometer via continuous flow isotope ratio mass spectrometry. The Colorado Plateau Stable Isotope Laboratory at Northern Arizona University analyzed carbon and nitrogen stable isotopes in hair using a Carlo Erba NC2100 elemental analyzer interfaced via a CONFLO III device to an isotope ratio monitoring mass spectrometer. The same lab measured sulfur stable isotopes using a DELTA plus Advantage IRMS configured through a CONFLO III using a Costech ECS4010 Elemental Analyzer (DELTA V Advantage/DELTA plus Advantage, Thermo-Scientific, Waltham, MA, USA).

We used conventional delta (*δ*) notation to report the differences of stable isotope ratios, expressed as *δ*^13^C, *δ*^15^N, and *δ*^34^S values (in parts per thousand, ‰), between samples and Vienna Peedee belemnite (VPDB), atmospheric N_2_ (Air), and Vienna Cañon Diablo Toilite (VCDT), respectively. The Colorado Plateau lab used the following international measurement standards: IAEA CH6 and IAEA CH7 for *δ*^13^C, IAEA N1 and IAEA N2 for *δ*^15^N, and IAEA S1-S4, IAEA SO5, and SO6 for *δ*^34^S. The Colorado Plateau lab estimated an analytical error (+1 SD) of ±0.2 ‰ for *δ*^13^C, ±0.3 ‰ for *δ*^15^N, and ±0.4 ‰ for *δ*^34^S. The Washington State lab used the following international measurement standards: USGS 40 and 41 for *δ*^13^C, USGS 32, 25, and 26 for *δ*^15^N, and IAEA S-2, S05, and S3 for *δ*^34^S. The Washington State lab estimated an analytical error (+1 SD) of ±0.1 ‰ for *δ*^13^C, ±0.2 ‰ for *δ*^15^N, and ±0.35 ‰ for *δ*^34^S.

#### Statistical analyses

We compared stable isotope values for male and female grizzly bears and conducted pair-wise comparisons between years. We used Shapiro-Wilk and Levene’s tests to determine if isotope values (mixture components) of each sex and year were normally distributed and homoscedastic following the assumptions of normal mixture models [27]. We then compared isotope values between both sexes and years using either ANOVA (normal distribution and equal variance) or Kruskal–Wallis tests (non-normal distribution and/or unequal variance). We also used *t*-tests to compare stable isotope values for grizzly bears sampled in Cooke City to those published in past studies from the GYE. We note, in most cases, that sample sizes and/or standard deviations were not given in past studies; therefore, we only conducted statistical comparisons of stable isotope values when possible. We conducted all significance tests with α = 0.05.

For our main analysis, we estimated the diets of grizzlies using a four-step approach (Box 1).

## Results

The genetics lab identified 18 grizzly (6M:12F) bears. Thirteen of the 18 (6M:12F) grizzlies were recaptured at least once during other years. We measured the stable isotope ratios in hair samples from 14 (4M:10F) of 18 individuals captured in 2007–2009 and considered three recaptured bears as independent (*n* = 17; Table 1). We did not conduct SIA on the hair of the remaining four individuals because guard hair samples for these bears were not available.

### Stable isotope values for grizzlies

We found that mean stable isotope values were similar between males and females, but different for *δ*^13^C and *δ*^34^S among years (Table 1). Grizzly bears sampled in Cooke City had different isotope values than those sampled throughout the GYE in the past. The mean *δ*^34^S values for grizzlies (hair) sampled in Cooke City were relatively low in 2007–2009 ($\bar{x}$ = 3.5±1.3 ‰) compared to those derived from hair and blood collected throughout the GYE in 1994–2001 ($\bar{x}$ ranged from ~4.0 to 7.8 ‰; [13]) and hair in 2000–2010 ($\bar{x}$ = 4.6±0.3 ‰ for adults; all bears: 4.4±1.5 ‰, *t* = -2.52, *df* = 25.9, *P* = 0.018; [14]). We also found that the mean *δ*^15^N values for bears sampled in Cooke City in 2007–2009 (hair: $\bar{x}$ = 3.1±1.1 ‰, range = 1.4,5.1 ‰) were relatively low compared to other time periods when bears were sampled throughout the GYE: 1977–96 (hair and bone with no access to human foods: $\bar{x}$ range = 5.5,7.8 ‰; [40]); 1994–2001 (hair and blood: $\bar{x}$ = 4.5 ‰; [13]); 2000–2010 (hair: $\bar{x}$ = 5.5±0.1 ‰; *t* = -9.03, *df* = 16.0, *P*<0.001; [12]); and Yellowstone Lake in 2007–2009 (hair: $\bar{x}$ = 5.1±1.6 ‰; *t* = -6.69, *df* = 25.5, *P*<0.001; [19]).

### Modeling grizzly bear diets

We estimated the diets of bears using four combinations of stable isotope values for grizzly bear hair and their foods (Fig 2). We found *δ*^13^C had the lowest NRMSE values, especially when paired with *δ*^15^N, suggesting *δ*^13^C had potentially more predictive power than other tracers (Table 2). We found that the concentration dependence models without group structure were the most parsimonious of the main models in each candidate set (lowest DIC and PED values) and that the *δ*^13^C/*δ*^15^N model without group structure (CN(CD) ∼ (1|Bear)) and year models (CN(CD) ∼ (1|Year) + (1|Bear)) were the top models overall (lowest DIC_rescaled_ and PED values) (Table 2 & S4 Table). The *δ*^13^C/*δ*^34^S model yielded biologically unreasonable dietary estimates (Table 3) because the *δ*^13^C and *δ*^34^S values for ungulates overlapped those for bears and were collinear with plants and whitebark pine seeds (Fig 2C). We did not use this set of stable isotope measurements for any further analyses. As expected, each null model had the highest DIC value and DIC for 4-source models were higher than the 3 main models (i.e., 3-source concentration dependence models; Table 2). We note that although 4-source models ranked poorly compared to our main models (Table 2), they yielded similar dietary estimates and confirmed that trout did not contribute (~0%) to the diets of bears (S3 Table).

**Table 2:** DIC model selection results for SIMMs used to estimate the diets of grizzly bears sampled in Cooke City Basin, Montana, 2007–2009.

Candidate set	Model	$\bar{D}$	DIC	ΔDIC	DIC_Rescaled_	ΔDIC_Rescaled_	NRMSE *δ*^13^C	NRMSE *δ*^15^N	NRMSE *δ*^34^S
*δ*^13^C/*δ*^15^N/*δ*^34^S	CNS ∼ (1|Bear)	1661.85	1689.60	925.30	1168.27	667.95	0.268	0.310	0.219
(*n* = 51)	CNS(CD) ∼ (1|Bear)	713.42	764.30	0.00	526.49	26.17	0.252	0.293	0.211
	CNS(CD) ∼ (1|Sex) + (1|Bear)	716.92	770.23	5.93	531.26	30.94	0.248	0.285	0.195
	CNS(CD) ∼ (1|Year) + (1|Bear)	713.96	772.80	8.50	534.81	34.49	0.267	0.358	0.194
	CNS(CD) ∼ (1|Bear), 4-source	747.78	815.39	51.09	566.13	65.81	0.260	0.292	0.213
	Mean						0.259	0.308	0.206
*δ*^13^C/*δ*^15^N	CNS ∼ (1|Bear)	771.26	794.84	294.52	794.84	294.52	0.281	0.304	
(*n* = 34)	CN(CD) ∼ (1|Bear)	462.11	500.32	0.00	500.32	***0*.*00***	0.151	0.297	
	CN(CD) ∼ (1|Sex) + (1|Bear)	465.46	505.85	5.53	505.85	5.53	0.139	0.302	
	CN(CD) ∼ (1|Year) + (1|Bear)	462.95	502.73	2.41	502.73	***2*.*41***	0.140	0.345	
	CN(CD) ∼ (1|Bear), 4-source	501.15	552.48	52.16	552.48	52.16	0.270	0.285	
	Mean						0.196	0.307	
*δ*^15^N/*δ*^34^S	NS ∼ (1|Bear)	1532.10	1556.78	298.67	1556.78	1056.46		0.290	0.226
(*n* = 34)	NS(CD) ∼ (1|Bear)	1219.68	1258.11	0.00	1258.11	757.79		0.283	0.217
	NS(CD) ∼ (1|Sex) + (1|Bear)	1220.29	1259.67	1.56	1259.67	759.35		0.290	0.197
	NS(CD) ∼ (1|Year) + (1|Bear)	1223.10	1260.93	2.82	1260.93	760.61		0.324	0.213
	NS(CD) ∼ (1|Bear), 4-source	1249.67	1301.84	43.73	1301.84	801.52		0.285	0.220
	Mean							0.294	0.215

**Table 3:** Proportional dietary contributions for grizzly bears sampled in Cooke City Basin, Montana, 2007–2009. Parameters were estimated by IsotopeR using concentration dependence models without group structure. *Indiv-level* denotes the Range of mean marginal posterior distributions for all individual bears.

	Pop-level		Credible interval			Indiv-level
Sources	$\bar{x}$ (%)	1 SD	2.5%	50%	97.5%	Range (%)
CNS(CD) ∼ (1|Bear)						
Plants	54.0	8.5	34.5	54.5	69.0	49, 60
Ungulates	14.2	9.5	1.9	12.3	40.8	13, 16
Whitebark	31.8	7.4	16.9	31.8	46.1	26, 36
CN(CD) ∼ (1|Bear)						
Plants	55.8	9.8	35.1	56.3	73.8	52, 59
Ungulates	9.5	7.7	0.0	8.8	24.3	9, 11
Whitebark	34.7	9.5	17.3	34.3	54.2	33, 39
SN(CD) ∼ (1|Bear)						
Plants	56.6	10.2	37.1	56.2	78.1	51, 64
Ungulates	6.9	5.9	0.0	6.1	20.3	6, 9
Whitebark	36.5	10.5	15.1	36.9	56.2	29, 41
CS(CD) ∼ (1|Bear)						
Plants	0.7	3.4	0.0	0.0	9.2	1, 2
Ungulates	98.3	5.7	80.8	100.0	100.0	95, 98
Whitebark	1.0	3.1	0.0	0.0	11.5	1, 4

The assimilated diets of grizzlies sampled in Cooke City Basin were largely composed of plants and whitebark pine seeds (Table 3). Females may have consumed more pine seeds than male bears prior to sampling (probability of similarity of mean posterior distributions = 0.63) and the consumption of seeds may have decreased through time (probability of similarity of mean posterior distributions in 2007 & 2009 = 0.47) (Table 4). In addition, the DIC_Rescaled_ values for the more complex 3-isotope models were higher than the 2-isotope models, suggesting that *δ*^13^C/*δ*^15^N/*δ*^34^S may not be the best choice for modeling the diets of bears without the inclusion of army cutworm moths or trout as a major diet source (Table 2). Instead, it would be more cost effective to model the diets of bears that do not use army cutworm moths or trout using *δ*^13^C/*δ*^15^N. Although the *δ*^13^C/*δ*^15^N concentration dependence model without group structure was the top model overall, we note that the other top models in each candidate set (also concentration dependence models without group structure) yielded similar mean proportional dietary contributions (Table 3, S5 Table). To our knowledge, this is the first study to demonstrate that different combinations of stable isotope values for the same consumers and their foods can yield similar dietary estimates when using SIMMs.

**Table 4:** Proportional dietary contributions for grizzly bears sampled in Cooke City Basin, Montana, 2007–2009. Parameters were estimated by IsotopeR using *δ*^13^C/*δ*^15^N concentration dependence models with group structure. These models were ranked #2 and #3 overall when comparing DIC_Rescaled_ values (Table 2).

				Credible interval		
Source	Sex/Yr	$\bar{x}$	1 SD	2.5%	50%	97.5%
Plants	F	56.4	11.3	32.9	56.6	32.9
	M	62.4	16.9	28.1	62.5	28.1
Ungulates	F	6.3	6.8	0.0	4.8	0.0
	M	7.0	8.8	0.0	4.0	0.0
Whitebark	F	37.3	10.9	17.8	36.7	17.8
	M	30.6	15.8	1.8	29.4	1.8
Plants	2007	48.1	17.6	11.6	48.7	82.0
	2008	51.5	16.1	17.1	52.2	81.9
	2009	60.5	11.1	38.7	60.4	82.6
Ungulates	2007	7.8	7.1	0.1	6.2	25.5
	2008	9.4	8.1	0.1	7.7	29.1
	2009	9.5	6.9	0.2	8.6	24.6
Whitebark	2007	44.1	17.7	12.2	42.8	83.1
	2008	39.1	15.5	12.1	37.7	73.6
	2009	30.0	10.4	10.6	29.5	51.5

## Discussion

We found that grizzly bears sampled in Cooke City Basin primarily consumed whitebark pine seeds and other plant foods prior to sampling. Meat was not a large contributor to the assimilated diets of grizzlies sampled in Cooke City; at least not during the time period associated with hair growth. Stable isotope values measured in bear hair collected elsewhere in the GYE and North America support our results. Below, we compare our stable isotope data to those from past studies and discuss SIMM results to make general conclusions about the diets of grizzly bears in Cooke City Basin prior to sampling each year in 2007–2009. We also put forward our new analytical tools and model selection approach for estimating the diets of grizzlies and other free-ranging animals using stable isotope data.

### Stable isotope values for grizzlies

We found that *δ*^13^C values (Fig 2) had potentially more predictive power than other stable isotope values when estimating the diets of grizzly bears in Cooke City Basin (Table 2). It is unclear if the *δ*^13^C values for bear tissues have changed through time in the GYE, as previous studies did not report those results. Although Felicetti et al. [13] considered using *δ*^13^C values to model the diets of bears, they found that *δ*^13^C values in captive bears (plasma) did not track dietary *δ*^13^C values as cleanly as *δ*^15^N and *δ*^34^S values. Therefore, they did not use the *∆*^13^C factors estimated in their study to adjust the *δ*^13^C values of bear foods in their isotopic mixing space. No study has used this classic stable isotope tracer (*δ*^13^C) to aid in understanding the assimilated (plant-based) diets of free-ranging grizzlies in the GYE. We found that the *∆*^13^C for rat hair was similar to those derived from the hair of other mammals [31]. Unlike most studies that use stable isotopes to estimate the diets of mammals, we added different *∆*^13^C factors [29] to the plant and animal food sources in our study. We found that after correcting for isotopic discrimination, the *δ*^13^C values for major food sources were isotopically distinct (Fig 2 and S1 Fig).

The *δ*^15^N values measured in the hair of grizzlies sampled in Cooke City Basin (Table 1) were lower than other grizzly populations in North America with primarily herbaceous diets consisting of C_3_ plants [41]. In addition, the *δ*^15^N values for bears sampled in Cooke City Basin in 2007–2009 were relatively low compared to other time periods when bears were sampled in the GYE. It is likely that bears sampled around the Cooke City Basin in 2007–2009 ate less meat, prior to sampling, than other North American grizzly populations and those sampled throughout the GYE during other time periods. It is possible that the *δ*^15^N values derived from the hair of bears sampled in Cooke City Basin were lower than those from Yellowstone Lake during the same time period [19] (suggesting less meat consumption in Cooke City Basin) because the *δ*^15^N values for hair represent different time periods. Bears in Cooke City Basin were sampled in September and October and samples from Fortin et al. [19] were collected from mid-May to mid-August (i.e., stable isotopes in hair represented bear diets from the previous year); therefore, meat potentially consumed in October and November would not have been fully assimilated into the hair of bears sampled in Cooke City Basin.

The *δ*^34^S values for Cooke City Basin grizzlies (hair) were relatively low in 2007–2009 compared to bears sampled throughout the GYE in 1994–2001 and 2000–2010. Isotopic data suggest that grizzlies sampled in Cooke City Basin may have consumed less whitebark, prior to sampling, than bears sampled during other time periods, or the contribution of whitebark may have declined in grizzly diets through time in the GYE. Similar to Schwartz et al. [14], we found that the mean *δ*^34^S values for bears decreased with increased whitebark pine mortality from 2007 to 2009. Although our sample sizes were small, such corroborating evidence (with [14]) suggest that grizzlies may have responded to a decrease in whitebark availability by consuming less seeds.

### Modeling grizzly bear diets

Except for the *δ*^13^C/*δ*^34^S model, the 2-source concentration dependence models with no group structure were the most parsimonious (lowest DIC and PED) models in each candidate set (Table 2 and S4 Table); each yielded comparable proportional dietary contributions (Table 3).

We recommend the use of *δ*^13^C/*δ*^15^N models to estimate the diets of grizzlies that did not have access to trout or moths. We also suggest that researchers consider the use of 3-isotope, 4-source models to estimate the diets of bears sampled around Yellowstone Lake. Researchers could use isotope data derived from sampled tissues to estimate the contribution of trout in the diets of bears through time. In addition, comparing the diets of bears that foraged on trout and those that did not access trout historically could provide researchers valuable insights related to the foraging ecology of grizzlies in the GYE. In addition to measuring trout in bear diets, we also recommend sampling moths more extensively and measuring their isotopic composition to better estimate their place in an isotopic mixing space.

Although we recognize the uncertainty associated with the digestibility of S (i.e., including *δ*^34^S in a model could bias parameter estimates if digestibility is not properly accounted for), we still recommend the use of the 3-isotope models to estimate the diets of grizzlies in the GYE when trout are available. We validate this suggestion by showing that the top *δ*^13^C/*δ*^15^N model (no *δ*^34^S) and 3-isotope model (with *δ*^34^S) yielded comparable parameter estimates (Table 3) and both models estimated trout at ~0% (S3 Table). We do, however, encourage controlled diet experiments that aim to estimate the digestibility of S in foods and discrimination factors (*∆*^34^S) for mammal tissues because the use of *δ*^34^S to investigate animal diets is gaining popularity.

Understanding grizzly bear responses to losses in major food sources is essential for predicting how the population will adapt to future environmental change. It is currently unclear how the diets of bears throughout the GYE have responded to declining whitebark pine, cutthroat trout, and ungulates. Results from prior studies suggest that whitebark pine is a primary food source for grizzlies [19,42], and perhaps more so for females than males [11,43] (Table 4). Both Schwartz et al. [12] and van Manen et al. [11] recently found that female body fat may have decreased from 2007 to 2009 when whitebark pine mortality increased along cone production transects [44]. Declining body fat for females [11,12] and declining *δ*^34^S values for bears ([14] and this study) suggests that bears may have consumed less whitebark pine seeds during years prior to sampling. Several recent studies suggest that grizzlies may consume more meat in response to losses of major food sources such as cutthroat trout [45], especially during years when whitebark pine seed production is low [12]. We found that meat contributions for bears in Cooke City Basin were relatively low compared to whitebark and other plants (Table 3). It seems plausible that some grizzly bears have responded to losses in whitebark by consuming more plant matter [1] (Table 4), including berries [46]. An increased consumption of berries and other plant matter by females seems sensible, as plants are more readily available and generally safer to forage for than meat [47]. In general, it seems reasonable to hypothesize that many females in the GYE continue to forage for whitebark pine seeds as a primary food source during hyperphagia (especially in Cooke City Basin; this study and [48]) and throughout the year when abundant, males consume more meat, and plant matter has increased in the diets of all bears. Although evidence suggests that the dietary responses of bears could have changed during the course of our study, such changes may be artifacts of a small sample size. We encourage researchers to use the methods described here to conduct a large-scale analysis of bear diets in the GYE. Modeling the diets of bears through time and space using SIMMs could aid in grizzly bear conservation in the GYE by providing definitive answers to many of the pressing questions related to resource use of bears in this rapidly changing environment.

SIMM analysis is gaining popularity among ecologists. To our knowledge, this is the first study to i) demonstrate that different combinations of stable isotope values for consumers and their foods can generate similar proportional dietary contributions using SIMMs; ii) use model selection to evaluate SIMMs (use R package “IsotopeR” [49]; download user guide and example data from http://jackhopkinswildlife.com/isotoper/); and iii) generate reliable estimates of whitebark pine seed consumption by grizzly bears in the GYE using stable isotope data. We hope researchers use our new statistical framework to reconstruct the diets of other free-ranging animals, as such analyses could be useful in answering many important, and often fundamental, questions in both basic and applied ecology.

## Supporting information

S1 Fig Rotating 3D plot of stable isotope values (top: *δ*^13^C, bottom: *δ*^15^N, vertical: *δ*^34^S) for hair of female (open circles) and male (closed circles) grizzly bears and their major bear foods (corrected for isotopic discrimination), including plants (red), ungulates (green), and whitebark pine seeds (blue), in Cooke City Basin, Montana, 2007–2009. (GIF)

S1 Table Hair-snare locations (UTMs) in Cooke City Basin, Montana, 2007–2009. (PDF)

S2 Table Discrimination-corrected stable isotope values and digestible elemental concentrations for major bear foods used to estimate the diets of grizzly bears in Cooke City Basin, Montana, 2007–2009. (PDF)

S3 Table Estimated proportional dietary contributions for grizzly bears sampled in Cooke City Basin, Montana in 2007–2009 using 4-source concentration dependence SIMMs with no random sex or time effects (CNS(CD) ∼ (1|Bear)). (PDF)

S4 Table PED model selection results for SIMMs used to estimate the diets of bears sampled in Cooke City Basin, Montana, 2007–2009. (PDF)

S5 Table Probability of similarity of mean marginal posterior distributions for different foods estimated by concentration dependence SIMMs with no random sex or time effects (top models in each candidate set). (PDF)
