# Supplementary figures and images for: Selecting the best stable isotope mixing model to estimate grizzly bear diets in the Greater Yellowstone Ecosystem
Source: PLoS One. 2017 May 11;12(5):e0174903. doi: 10.1371/journal.pone.0174903 (PMC5426898; doi:10.1371/journal.pone.0174903)

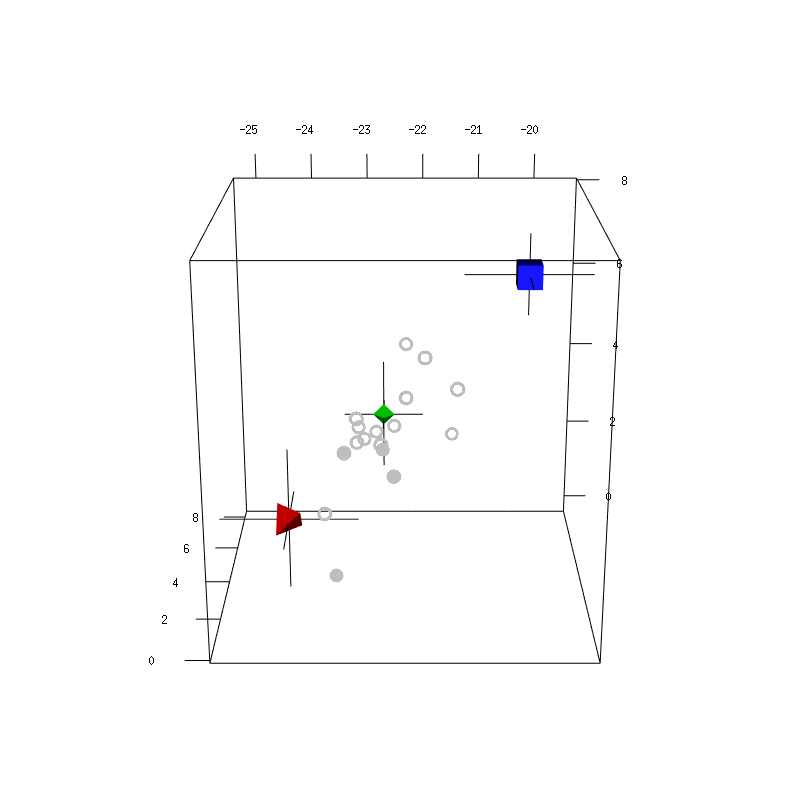

Supplement: S1 Fig — (GIF) [file pone.0174903.s001.gif]
