# Supplementary material for: Selecting the best stable isotope mixing model to estimate grizzly bear diets in the Greater Yellowstone Ecosystem
Source: PLoS One. 2017 May 11;12(5):e0174903. doi: 10.1371/journal.pone.0174903 (PMC5426898; doi:10.1371/journal.pone.0174903)
Supplement: S1 Table — (PDF) [file pone.0174903.s002.pdf]

S1 Table. Hair-snare locations (UTMs) in Cooke City Basin, Montana, 2007–2009.

Locations	Easting	Northing
Fisher Creek	584268	4989786
Miller Creek	583797	4987318
Republic Creek	583768	4983319
Sheep Creek	580375	4986850
Woody Creek	586408	4985484
Mud Lake	586444	4991119
Still Water	581552	4990817
Cooke City Face	584625	4986762
