# Supplementary material for: Selecting the best stable isotope mixing model to estimate grizzly bear diets in the Greater Yellowstone Ecosystem
Source: PLoS One. 2017 May 11;12(5):e0174903. doi: 10.1371/journal.pone.0174903 (PMC5426898; doi:10.1371/journal.pone.0174903)
Supplement: S2 Table — (PDF) [file pone.0174903.s003.pdf]

S2 Table. Discrimination-corrected stable isotope values and digestible elemental concentrations for major bear foods used to estimate the diets of grizzly bears in Cooke City Basin, Montana, 2007–2009.

Digest [N] & [C] = Digest N or C/Digest DM * 100
Digest N = Digest protein * 0.16 OR Digest N = %N*1 for animals and 0.9 for plants
Digest C = determined by Phillips and Koch (2002)
Digest DM = see Lopez-Alfaro 2015
Digest protein = crude protein * 1.0 for meat or 0.9 for veg and fruit (Koch and Phillips 2003)
Crude protein = %N * 6.25

Bear food	Raw stable isotope values			Stable isotope values corrected for isotopic discrimination				Digestible elemental concentrations	
	$\delta^{13}C$	$\delta^{15}N$	$\delta^{34}S$	$\delta^{13}C$	$\delta^{15}N$	$\delta^{34}S$-corr	$\delta^{34}S$	[C]	[N]
Whitebark pine seeds (*n* = 31)									
Pinus albicaulis	-22.4	-0.8	7.8	-19.0	1.6	-0.9	6.9	61.9	8.9
Pinus albicaulis	-24.1	-2.4	6.6	-20.7	0.0	-0.6	6.1	61.9	6.9
Pinus albicaulis	-23.0	0.0	7.6	-19.6	2.4	-0.8	6.8	61.9	7.5
Pinus albicaulis	-25.9	-1.0	8.7	-22.5	1.4	-1.1	7.6	61.9	7.2
Pinus albicaulis	-23.3	2.3	9.1	-19.9	4.7	-1.2	7.9	61.9	8.2
Pinus albicaulis	-25.2	-1.3	8.1	-21.8	1.1	-1.0	7.2	61.9	4.6
Pinus albicaulis	-23.7	-0.2	8.2	-20.3	2.2	-1.0	7.3	61.9	7.6
Pinus albicaulis	-24.2	-1.4	7.5	-20.8	1.0	-0.8	6.7	61.9	7.4
Pinus albicaulis	-25.8	-1.5	7.4	-22.4	0.9	-0.8	6.7	61.9	6.0
Pinus albicaulis	-23.5	-2.1	7.3	-20.1	0.3	-0.7	6.5	61.9	7.0
Pinus albicaulis	-24.0	-1.2	8.7	-20.6	1.2	-1.1	7.6	61.9	7.7
Pinus albicaulis	-23.8	1.7	8.9	-20.4	4.1	-1.2	7.7	61.9	6.4
Pinus albicaulis	-23.2	2.3	8.4	-19.8	4.7	-1.0	7.4	61.9	6.9
Pinus albicaulis	-22.7	2.0	8.3	-19.3	4.4	-1.0	7.3	61.9	7.9
Pinus albicaulis	-23.6	-1.0	8.4	-20.2	1.4	-1.0	7.4	61.9	7.2
Pinus albicaulis	-24.1	2.4	8.2	-20.7	4.8	-1.0	7.2	61.9	7.1
Pinus albicaulis	-24.2	0.6	9.3	-20.8	3.0	-1.3	8.0	61.9	7.9
Pinus albicaulis	-23.4	0.6	8.1	-20.0	3.0	-1.0	7.2	61.9	6.6
Pinus albicaulis	-22.4	0.6	8.1	-19.0	3.0	-1.0	7.2	61.9	8.3

	Pinus albicaulis	-23.8	4.5	9.8	-20.4	6.9	-1.4	8.4	61.9	7.9
	Pinus albicaulis	-22.1	-0.9	7.7	-18.7	1.5	-0.8	6.8	61.9	6.4
	Pinus albicaulis	-23.2	2.7	9.3	-19.8	5.1	-1.3	8.0	61.9	6.8
	Pinus albicaulis	-22.0	1.2	7.6	-18.6	3.6	-0.8	6.8	61.9	7.7
	Pinus albicaulis	-22.5	1.1	8.3	-19.1	3.5	-1.0	7.3	61.9	8.2
	Pinus albicaulis	-25.4	1.0	5.2	-22.0	3.4	-0.2	5.0	61.9	7.2
	Pinus albicaulis	-26.1	1.0	5.7	-22.7	3.4	-0.3	5.3	61.9	7.9
	Pinus albicaulis	-23.9	2.0	6.4	-20.5	4.4	-0.5	5.9	61.9	6.7
	Pinus albicaulis	-23.7	-1.2	8.4	-20.3	1.2	-1.0	7.4	61.9	7.2
	Pinus albicaulis	-23.5	0.1	7.9	-20.1	2.5	-0.9	7.0	61.9	6.7
	Pinus albicaulis	-23.7	3.4	8.4	-20.3	5.8	-1.0	7.4	61.9	7.9
	Pinus albicaulis	-23.4	2.1	8.1	-20.0	4.5	-0.9	7.1	61.9	6.8
	Mean	-23.7	0.5	8.0	-20.3	2.9		7.1		
	1 SD	1.1	1.7	1.0	1.1	1.7		0.7		
Plants (*n* = 43)										
bearberry	*Arctostaphylos uva-ursi*	-28.9	-7.8	-0.7	-25.5	-5.4	1.3	0.7	45	0.6
strawberry	Frageria	-26.8	-1.1	0.4	-23.4	1.3	1.1	1.4	45	1.8
currant	Ribes	-26.5	-3.3	4.7	-23.1	-0.9	-0.1	4.6	45	1.7
huckleberry	*Vaccinium* spp	-27.0	-2.9	1.4	-23.6	-0.5	0.8	2.2	45	0.7
huckleberry	*Vaccinium* spp	-26.2	-4.5	2.3	-22.8	-2.1	0.6	2.9	45	0.7
whortleberry	*Vaccinium* spp	-28.4	-4.5	3.3	-25.0	-2.1	0.3	3.6	45	1.3
spring beauty	*Claytonia lanceolata*	-28.6	-2.4	3.0	-25.2	0.0	0.4	3.4	45	6.1
angelica	Angelica	-27.4	-1.0	-2.5	-24.0	1.4	1.8	-0.7	45	8.9
spring beauty	*Claytonia lanceolata*	-27.3	0.1	3.5	-23.9	2.5	0.3	3.7	45	9
fire-weed	Epilobium	-29.1	-1.3	3.3	-25.7	1.1	0.3	3.6	45	3.4
glacier lily	*Erythronium grandiflorum*	-24.8	3.7	-0.6	-21.4	6.1	1.3	0.7	45	9.3
cow parsnip	*Heracleum lanatum*	-26.8	0.3	-2.3	-23.4	2.7	1.8	-0.5	45	14
fern-leaf lovage	*Ligusticum filicinum*	-27.5	-1.4	4.5	-24.1	1.0	0.0	4.5	45	10.3
dandelion	Taraxacum	-29.4	0.8	-2.4	-26.0	3.2	1.8	-0.6	45	7.1
elk thistle	*Cirsium Scariosum*	-27.5	-0.2	3.6	-24.1	2.2	0.2	3.8	45	10
Spring beauty	*Claytonia lanceolata*	-28.2	0.8	3.4	-24.8	3.2	0.3	3.7	45	11.6
glacier lily	*Erythronium grandiflorum*	-26.6	-2.1	3.2	-23.2	0.3	0.3	3.5	45	8.7
cow parsnip	*Heracleum lanatum*	-27.2	1.7	-1.2	-23.8	4.1	1.5	0.2	45	12.4
fern-leaf lovage	*Ligusticum filicinum*	-28.3	-2.1	5.4	-24.9	0.3	-0.2	5.2	45	7.8
licorice root	Osmorhiza	-30.2	0.4	1.3	-26.8	2.8	0.8	2.1	45	13.7

dandelion	Taraxacum	-28.6	0.2	-3.0	-25.2	2.6	1.9	-1.1	45	6.1
clover	Trifolium	-28.9	-1.7	11.6	-25.5	0.7	-1.8	9.7	45	9.5
biscuit root	Lomatium	-28.4	-0.3	2.7	-25.0	2.2	0.5	3.2	45	9.9
licorice root	Osmorhiza	-28.6	-5.0	2.3	-25.2	-2.6	0.6	2.8	45	1.7
elk thistle	*Cirsium scariosum*	-27.2	1.4	-7.7	-23.8	3.8	3.2	-4.5	45	3.2
elk thistle	*Cirsium scariosum*	-26.8	1.4	4.8	-23.4	3.8	-0.1	4.7	45	4.7
glacier lily	*Erythronium grandiflorum*	-24.8	2.5	-1.1	-21.4	4.9	1.5	0.3	45	2.3
biscuit root	Lomatium	-28.1	-2.9	0.2	-24.7	-0.5	1.1	1.3	45	1.4
elk thistle	*Cirsium scariosum*	-26.8	6.3	2.1	-23.4	8.7	0.6	2.7	45	3.2
cow parsnip	*Heracleum lanatum*	-25.4	-0.7	-3.0	-22.0	1.7	1.9	-1.0	45	4.3
angelica	Angelica	-27.3	-0.9	-2.3	-23.9	1.5	1.8	-0.5	45	4.1
carex	Carex	-26.5	3.5	-5.3	-23.1	5.9	2.5	-2.7	45	6.7
horsetail	*Equisetum arvense*	-29.2	4.1	0.0	-25.8	6.5	1.2	1.2	45	5.2
yampa	*Pedicularis* spp.	-30.2	-6.6	4.2	-26.8	-4.2	0.1	4.3	45	3.3
fire-weed	Epilobium	-28.7	-1.4	3.5	-25.3	1.0	0.2	3.8	45	7.8
yampa	*Perideridia gairdnerii*	-26.1	2.1	-1.6	-22.7	4.5	1.6	0.0	45	2.2
chives	*Allium* spp.	-26.9	-0.6	2.2	-23.5	1.8	0.6	2.8	45	6.3
sticky geranium	*Geranium viscosissimum*	-26.7	0.9	4.1	-23.3	3.3	0.1	4.2	45	6.4
graminoid	Agropyron	-26.9	-0.8	2.7	-23.5	1.6	0.5	3.2	45	4
graminoid	Elymus	-28.8	-2.8	2.2	-25.4	-0.4	0.6	2.8	45	4.7
brachted lousewort	*Pedicularis* spp.	-29.6	-0.2	4.4	-26.2	2.2	0.0	4.4	45	8.2
graminoid	Phleum	-27.5	1.6	0.1	-24.1	4.0	1.1	1.2	45	3.8
graminoid	Poa	-26.3	0.6	6.0	-22.9	3.0	-0.4	5.6	45	3
	Mean	-27.6	-0.6	1.5	-24.2	1.8		2.2		
	1 SD	1.3	2.8	3.4	1.3	2.8		2.5		
Ungulates (*n* = 8)										
bison	*Bison bison*	-24.6	4.4	1.6	-22.5	8.3	0.8	2.3	51.5	16.2
bison	*Bison bison*	-24.2	3.5	-0.7	-22.1	7.4	1.3	0.6	51.5	14.6
elk	*Cervus elaphus*	-25.2	2.6	5.2	-23.1	6.5	-0.2	5.0	51.5	15.3
elk	*Cervus elaphus*	-24.9	5.1	3.0	-22.8	9.0	0.4	3.4	51.5	15.1
mule deer	*Odocoileus hemionus*	-24.1	4.0	4.3	-22.0	7.9	0.0	4.3	51.5	16.1
mule deer	*Odocoileus hemionus*	-24.9	2.2	4.6	-22.8	6.1	0.0	4.5	51.5	15.8
mule deer	*Odocoileus hemionus*	-25.6	2.0	0.2	-23.5	5.9	1.1	1.3	51.5	15
mule deer	*Odocoileus hemionus*	-25.0	4.0	-0.2	-22.9	7.9	1.2	1.0	51.5	15.1

		Mean	-24.8	3.5	2.2	-22.7	7.4		2.8		
		1 SD	0.5	1.1	2.3	0.5	1.1		1.7		
Cutthroat trout (*n* = 6)											
	Oncorhynchus clarki		-21.2	6.7	2.0	-19.1	10.6	0.7	2.6	54.8	14.4
	Oncorhynchus clarki		-18.9	7.0	0.5	-16.8	10.9	1.0	1.5	54.8	12.5
	Oncorhynchus clarki		-20.8	7.0	0.2	-18.7	10.9	1.1	1.3	54.8	5.6
	Oncorhynchus clarki		-15.4	7.2	0.6	-13.3	11.1	1.0	1.6	54.8	14.1
	Oncorhynchus clarki		-16.5	7.9	0.5	-14.4	11.8	1.0	1.5	54.8	12.3
	Oncorhynchus clarki		-22.2	6.9	0.7	-20.1	10.8	1.0	1.7	54.8	10.8
		Mean	-19.2	7.1	0.7	-17.1	11.0		1.7		
		1 SD	2.7	0.4	0.6	2.7	0.4		0.5		
