# Supplementary material for: Selecting the best stable isotope mixing model to estimate grizzly bear diets in the Greater Yellowstone Ecosystem
Source: PLoS One. 2017 May 11;12(5):e0174903. doi: 10.1371/journal.pone.0174903 (PMC5426898; doi:10.1371/journal.pone.0174903)
Supplement: S3 Table — (PDF) [file pone.0174903.s004.pdf]

S3 Table. Estimated proportional dietary contributions for grizzly bears sampled in Cooke City Basin, Montana in 2007–2009 using 4-source concentration dependence SIMMs with no random sex or time effects ($\mathrm{CNS(CD)} \sim (1|\mathrm{Bear})$).

Source	Pop-level		Credible interval		
	$\bar{x}$	1 SD	2.5%	50%	97.5%
δ^{13}C/δ^{15}N/δ^{34}S					
Plants	55.1	9.0	36.4	55.4	71.5
Trout	0.9	1.7	0.0	0.0	6.3
Ungulates	12.0	10.4	0.0	10.8	36.0
Whitebark	32.1	8.0	15.8	32.2	47.4
δ^{13}C/δ^{15}N					
Plants	57.3	9.9	37.2	57.5	76.2
Trout	0.8	1.5	0.0	0.2	5.6
Ungulates	8.4	6.4	0.1	7.8	23.0
Whitebark	33.5	9.1	16.6	33.1	52.7
δ^{34}S/δ^{15}N					
Plants	56.7	10.5	36.2	56.6	77.9
Trout	0.8	2.0	0.0	0.0	7.7
Ungulates	5.0	5.9	0.0	2.7	19.0
Whitebark	37.4	10.9	14.5	37.8	57.6
