# Supplementary material for: Selecting the best stable isotope mixing model to estimate grizzly bear diets in the Greater Yellowstone Ecosystem
Source: PLoS One. 2017 May 11;12(5):e0174903. doi: 10.1371/journal.pone.0174903 (PMC5426898; doi:10.1371/journal.pone.0174903)
Supplement: S4 Table — (PDF) [file pone.0174903.s005.pdf]

S4 Table. PED model selection results for SIMMs used to estimate the diets of bears sampled in Cooke City Basin, Montana, 2007–2009.

Candidate set	Model	*PED*	*ΔPED*	Rescaled *PED*	Rescaled *ΔPED*
$\delta^{13}C/\delta^{15}N/\delta^{34}S$	No C-D (Null)	1722.23	895.58	1168.28	621.39
	C-D	826.65	0.00	588.84	41.95
	C-D, Sex	836.30	9.65	597.32	50.43
	C-D, Year	844.36	17.72	606.38	59.49
	C-D, 4-source Mean	904.05	77.40	654.79	107.90
$\delta^{13}C/\delta^{15}N$	No C-D (Null)	823.17	276.28	823.17	276.28
	C-D	546.89	0.00	546.89	0.00
	C-D, Sex	554.94	8.06	554.94	8.06
	C-D, Year	551.91	5.02	551.91	5.02
	C-D, 4-source Mean	618.82	71.93	618.82	71.93
$\delta^{15}N/\delta^{34}S$	No C-D (Null)	1586.50	281.87	1586.50	1039.61
	C-D	1304.63	0.00	1304.63	757.74
	C-D, Sex	1307.59	2.96	1307.59	760.70
	C-D, Year	1308.57	3.94	1308.57	761.69
	C-D, 4-source Mean	1371.49	66.86	1371.49	824.60
