# Supplementary material for: Selecting the best stable isotope mixing model to estimate grizzly bear diets in the Greater Yellowstone Ecosystem
Source: PLoS One. 2017 May 11;12(5):e0174903. doi: 10.1371/journal.pone.0174903 (PMC5426898; doi:10.1371/journal.pone.0174903)
Supplement: S5 Table — (PDF) [file pone.0174903.s006.pdf]

S5 Table. Probability of similarity of mean marginal posterior distributions for different foods estimated by concentration dependence SIMMs with no random sex or time effects (top models in each candidate set).

Models	Sources	Probability of similarity
CNS vs. CN		
	Plants	0.89
	Ungulates	0.70
	Whitebark	0.81
CNS vs. SN		
	Plants	0.86
	Ungulates	0.48
	Whitebark	0.69
CN vs. SN		
	Plants	1.00
	Ungulates	0.79
	Whitebark	0.88
